# Melatonin regulates mitochondrial function to alleviate ferroptosis through the MT2/Akt signaling pathway in swine testicular cells

**DOI:** 10.1038/s41598-024-65666-1

**Published:** 2024-07-02

**Authors:** Yuanjie Zhao, Ge Qin, Biao Jiang, Jinglei Huang, Shiwen He, Hui Peng

**Affiliations:** 1 School of Tropical Agriculture and Forestry, Hainan University, Haikou, 570228 Hainan China; 2 College of Life and Health, Hainan University, Haikou, 570228 China; 3 College of Animal Science and Technology, Southwest University, Chongqing, 404100 China

**Keywords:** Melatonin, Testicular ferroptosis, Melatonin receptors, Akt signaling pathway, Cell biology, Cell death, Cell signalling

## Abstract

Increasing evidence has shown that many environmental and toxic factors can cause testicular damage, leading to testicular ferroptosis and subsequent male reproductive disorders. Melatonin is a major hormone and plays an vital role in regulating male reproduction. However, there is a lack of research on whether Mel can alleviate testicular cell ferroptosis and its specific mechanism. In this study, the results indicated that Mel could enhance the viability of swine testis cells undergoing ferroptosis, reduce LDH enzyme release, increase mitochondrial membrane potential, and affect the expression of ferroptosis biomarkers. Furthermore, we found that melatonin depended on melatonin receptor 1B to exert these functions. Detection of MMP and ferroptosis biomarker protein expression confirmed that MT2 acted through the downstream Akt signaling pathway. Moreover, inhibition of the Akt signaling pathway can eliminate the protective effect of melatonin on ferroptosis, inhibit AMPK phosphorylation, reduce the expression of mitochondrial gated channel (VDAC2/3), and affect mitochondrial DNA transcription and ATP content. These results suggest that melatonin exerts a beneficial effect on mitochondrial function to mitigate ferroptosis through the MT2/Akt signaling pathway in ST cells.

## Introduction

As one of the most criticaltransition metals, iron plays multiple functions in the body and participates in various physiological regulatory processes, while an imbalance in iron metabolism and redox homeostasis of the body can trigger ferroptosis. Ferroptosis differs from cell apoptosis, autophagy, pyroptosis, necrosis and other forms of death. It is manifested by unchanged nuclei, nonruptured plasma membranes, crinkled mitochondrial cristae, and a decrease in cellular mitochondrial membrane potential and is often accompanied by the accumulation of iron and the onset of lipid peroxidation^1,2^.

Ferroptosis has been suggested to play a role in male reproductive disorders, and a variety of environmental factors can contribute to the occurrence of ferroptosis and, consequently, male infertility. Studies have shown that smoking causes high levels of ferroptosis in seminal plasma and affects semen quality^3^. Particulate Matter 2.5 μm (PM_2.5_) causes inhibition of Sertoli cell proliferation and dysfunction through ferroptosis^4^. Furthermore, exposure to toxicants can cause testicular damage through ferroptosis. Arsenite damages mouse testicular tissue and triggers ferroptosis in testicular cells^5^. The involvement of ferroptosis has also been confirmed in arsenite-induced oligospermia in mice^6^. Cadmium (Cd) causes testosterone deficiency through ferroptosis, ultimately reducing testosterone production^7^. In addition, oxygen–glucose deprivation and reoxygenation in Sertoli cells also induce ferroptosis and lead to testicular damage^8^. Previous studies have demonstrated that ferroptosis leads to male reproductive disorders and is primarily due to testicular damage caused by ferroptosis.

Melatonin (N-acetyl-5-methoxytryptamine, Mel) is synthesized primarily in the pineal gland at night. Still it is produced in all other cell mitochondria in a noncircadian manner, and less than 5% of the total melatonin production occurs in the pineal gland. Melatonin can regulate pathological cell metabolism and mitochondrial function in circadian rhythm ^9^. It also regulates mitochondrial homeostasis, reduces DNA damage, and prevents the onset of apoptosis in the mitochondrial pathway^10,11^. In male reproduction, Mel reduces apoptosis of germ cells and restores testosterone secretion in the testis^12^. Mel can regulate the secretory function of Leydig and Sertoli cells, and it has also been suggested that Mel may promote sperm quality and viability during storage^13^. In recent years, studies have also linked the protective effects of Mel to ferroptosis. Mel can improve the osteogenic ability and reduce ferroptosis of MC3T3-E1 cells^14^. It also counteracts doxorubicin-induced cardiotoxicity by modulating the Yes-associated protein (YAP) and regulates ferroptosis^15^. It has also been shown that Mel attenuates lipid peroxidation in brain tissue, thereby regulating ferroptosis^16^. We, therefore, hypothesize that Mel may also protect against testicular damage caused by ferroptosis.

In the present study, we demonstrated for the first time the protective effect of Mel against ferroptosis in swine testis (ST) cells via melatonin receptor 1B (MT2) and further elucidated the regulation of the Akt signaling pathway downstream of MT2. Inhibition of the Akt signaling pathway affected the phosphorylation of AMPK and mitochondrial function, suggesting that the specific mechanism by which Mel regulates ferroptosis is through the MT2/Akt signaling pathway in ST cells.

## Results

### Melatonin protected ST cells against erastin-induced ferroptosis

**Figure 1:**
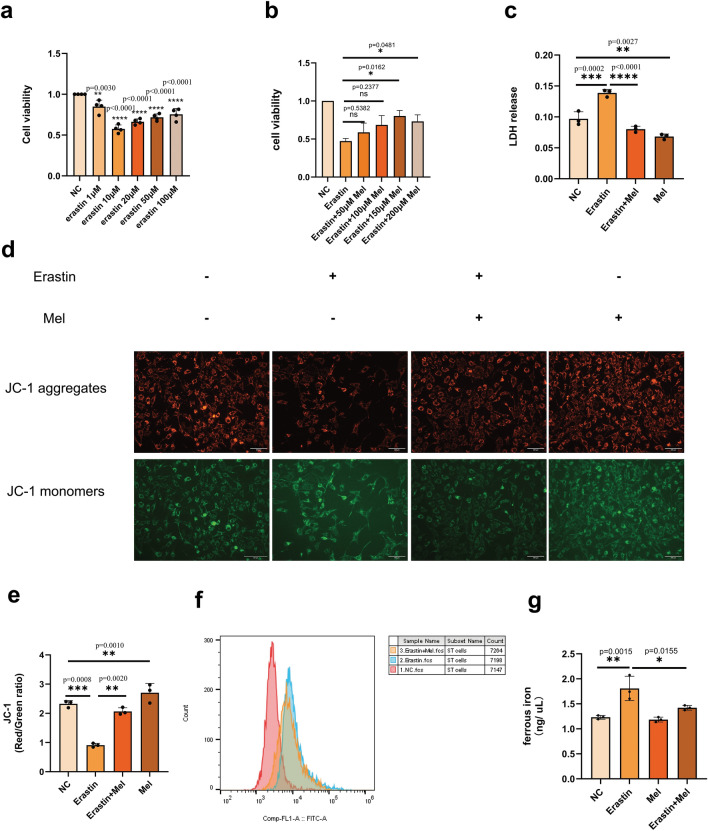
The effects of melatonin treatment on erastin-induced ferroptosis of ST cells. (**a**) MTT assay was used to detect the cell viability to select the optimal concentration of erastin. All erastin groups were compared with the NC group. Each group had three biological replicates. (**b**) Screening for concentrations of erastin and Melatonin coculture by cell viability using MTT assey, all groups were compared with the erastin treated group. Each group had three biological replicates. (**c**) Lactate dehydrogenase release were performed at 1 day after erastin treated and then 1 day of melatonin treated. The OD value was used to determine the amount of LDH enzyme released. Each group had three biological replicates. (**d**) Mitochondrial membrane potential was detected at 1 day after erastin-induced and then 1 day after melatonin treated. Red fluorescence shows cells with decreased membrane potential, and green fluorescence shows cells with normal membrane potential. Three fields were randomly collected from each group, and each group had three biological replicates. (**e**) Red/Green ratio of JC-1 staining. Mean fluorescence intensity was calculated using imagej. The mean fluorescence of three biological replicates per group were used for statistical analysis using mean fluorescence intensity. (**f**) ROS levels in ST cells were analyzed by flow cytometry. Cells were divided into three groups: NC, erastin and erastin + Mel. All *P* values are annotated on the graph. (**g**) Ferrous iron content assays were performed at 1 day after erastin treated and then 1 day after melatonin treated. Each group had three biological replicates. All *P* values are annotated on the graph. *P < 0.05, **P < 0.01, and ***P < .001.

Erastin is a ferroptosis inducer, mainly related to ROS and iron-dependent signaling. To investigate the effect of melatonin on erastin-induced ferroptosis, we used different concentrations of erastin to treat ST cells and then assayed cell viability by the MTT method. The results showed that cell viability was significantly reduced by 10 µM erastin treatment (Fig. 1a), while 150 µM Mel treatment for 24 h significantly increased cell viability after erastin induction (Fig. 1b). In subsequent experiments, we selected 10 µM erastin treatment for 24 h followed by 150 µM Mel treatment for 24 h as the working concentration and processing time, respectively.

To study the alleviating effect of Mel on ferroptosis, we examined lactate dehydrogenase (LDH) enzyme release in ST cells, and the results showed that cellular LDH release was sharply increased after erastin-induced ferroptosis and that there was a significant decrease in LDH enzyme content after the addition of Mel (Fig. 1c). Then, we examined the changes in MMP, and the ratio of red to green fluorescence values indicated that Mel treatment alleviated the decrease in cellular MMP caused by erastin (Fig. 1d,e). Studies have shown that erastin induced ferroptosis involves iron accumulation, leading to the generation of ROS^17^. To investigate whether melatonin allevistes erastin induced ST cells damage caused by ferroptosis, we then measured intracellular ROS levels, which increased after erastin induced ferroptosis and decreased after melatonin treatment (Fig. 1f). Next, we detected the intracellular ferrous iron content and found that the ferrous iron significantly increased after erastin induction, and decreased after the addition of exogenous melatonin (Fig. 1g). In summary, Mel decreases LDH enzyme release, ferrous iron content and ROS levels, increases MMP under erastin-supplemented conditions in ST cells.

**Figure 2:**
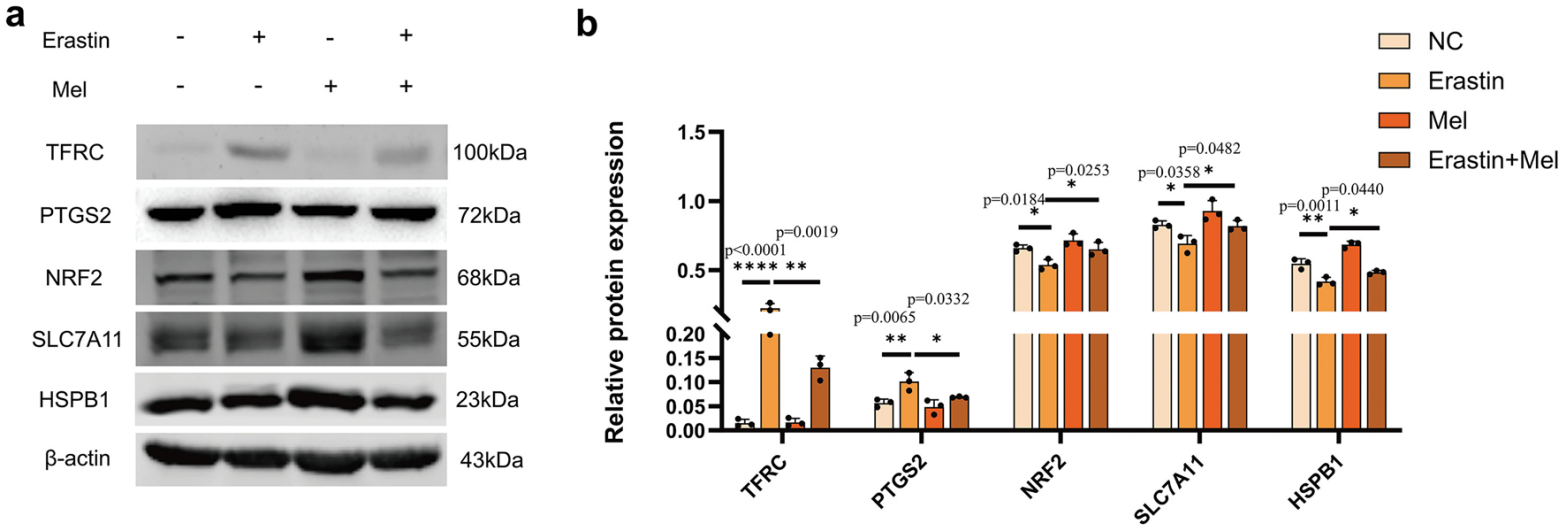
Melatonin affect ferroptosis related protein expression in ST cells. (**a**) Western blot for TFRC, PTGS2, NRF2, SLC7A11, HSPB1 and β-actin were performed. ST cells were divided into four groups: negative control(NC) , erastin, melatonin and erastin + melatonin, each group had three biological replicates. (**b**) Gray value analysis of TFRC, PTGS2, NRF2, SLC7A11, HSPB1. Statistical analysis between NC, erastin + Mel groups and Erastin groups were analyzed using one-way ANOVA with three biological replicates per group. All *P* values are annotated on the graph. *P < 0.05, **P < 0.01, and ***P < .001, *ns*, not significant.

To pursue the role of Mel in ferroptosis, we investigated the changes in the expression of five ferroptosis biomarkers, TFRC, PTGS2, NRF2, SLC7A11, and HSPB1 (Fig. 2a). The expression of TFRC and PTGS2 was evidently upregulated, while that of NRF2, SLC7A11 and HSPB1 was markedly downregulated (Fig. 2b). The above five protein trends are all consistent with previously published studies^18–22^. These results further demonstrated that Mel could mitigate erastin-induced cellular ferroptosis in ST cells.

### Melatonin alleviates ferroptosis in ST cells via the MT2 receptor

To further investigate how Mel alleviates erastin-induced ferroptosis in ST cells, we used different agonists and inhibitors targeting MT1 and MT2. We added 6-chloromelation and luzindole, the agonists and inhibitors of MT1, and 4-P-PDOT and 8-M-PDOT, the agonists and inhibitors of MT2, respectively. MMP and ferroptosis markers were measured to determine the receptor through which melatonin affects ferroptosis. In all subsequent experiments, inhibitors were added half an hour prior to Mel addition, and agonists were added at the same time as Mel.

**Figure 3:**
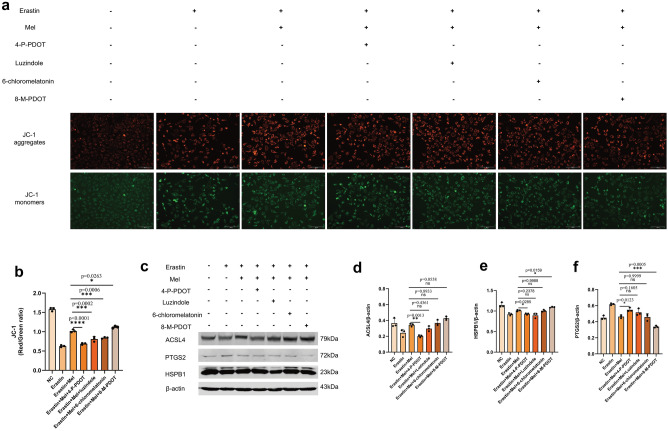
MT2 was involved in melatonin’s protection against erastin-induced ferroptosis. (**a**) MMP were measured on ST cells. Cells were divided into seven groups: negative control, erastin, erastin + melatonin, erastin + melatonin + 4-P-PDOT, erastin + melatonin + luzindole, erastin + melatonin + 6-chloromelatonin and erastin + melatonin + 8-M-PDOT. Three fields were randomly collected from each group, and each group had three biological replicates. (**b**) Red/Green ratio of JC-1 staining. Mean fluorescence intensity was calculated by imagej. Statistical analysis between erastin + melatonin and other groups were using one-way ANOVA. (**c**) Western blot for ACSL4, PTGS2, HSPB1 and β-actin were performed. ST cells were divided into seven groups same as A. Each group had three biological replicates. (**d**–**f**) Gray value analysis of ACSL4, PTGS2 and HSPB1. Statistical analysis between erastin + melatonin and other groups were using one-way ANOVA. All *P* values are annotated on the graph. *P < 0.05, **P < 0.01, and ***P < .001, *ns*, not significant.

First, we detected the changes in MMP using a JC-1 fluorescent probe (Fig. 3a). After analyzing the red‒green fluorescence ratio, we found that the MMP decreased significantly after the addition of 4-P-PDOT or luzindole inhibitors, while the addition of 8-M-PDOT to the culture medium significantly increased the MMP (Fig. 3b). Since 4-P-PDOT and 8-M-PDOT are potent inhibitors and agonists of MT2, the significant differences exhibited after the addition of these two drugs suggest that Mel alleviates cellular ferroptosis via the MT2 receptor. We further chose three ferroptosis biomarkers, ACSL4, PTGS2 and HSPB1, and studied their expression trends after treatment with agonists and inhibitors targeting Mel receptors. The results indicated that the expression of PTGS2 obviously increased, and the expression of ACSL4 and HSPB1 markedly decreased after inhibition of the MT2 receptor by 4-P-PDOT, while the expression of PTGS2 was significantly decreased, and the expression of ACSL4 and HSPB1 was markedly increased after 8-M-PDOT treatment to activate the MT2 receptor (Fig. 3c–f). The results further demonstrated that Mel eased ferroptosis in ST cells via the MT2 receptor.

### Mel protects ST cells from ferroptosis via the Akt signaling pathway

Some studies have shown that MT2 plays a role in different cells and tissues through downstream signaling pathways, so we selected the downstream pathways of the MT2 receptor for further investigation, which are the Akt^23^, CaMKII^24^, ERK^25^, JNK^26^, PKC^27^, mTOR^28^ and p38 MAPK^29^ signaling pathways. We then utilized MK2206, an inhibitor of Akt; KN-93, an inhibitor of CaMKII; FR180204, an inhibitor of ERK1/2; SP600125, an inhibitor of JNK; Ro31-8220, an inhibitor of PKC; AZD8055, an inhibitor of mTOR; and SB203580, an inhibitor of p38 MAPK for the follow-up experiments.

**Figure 4:**
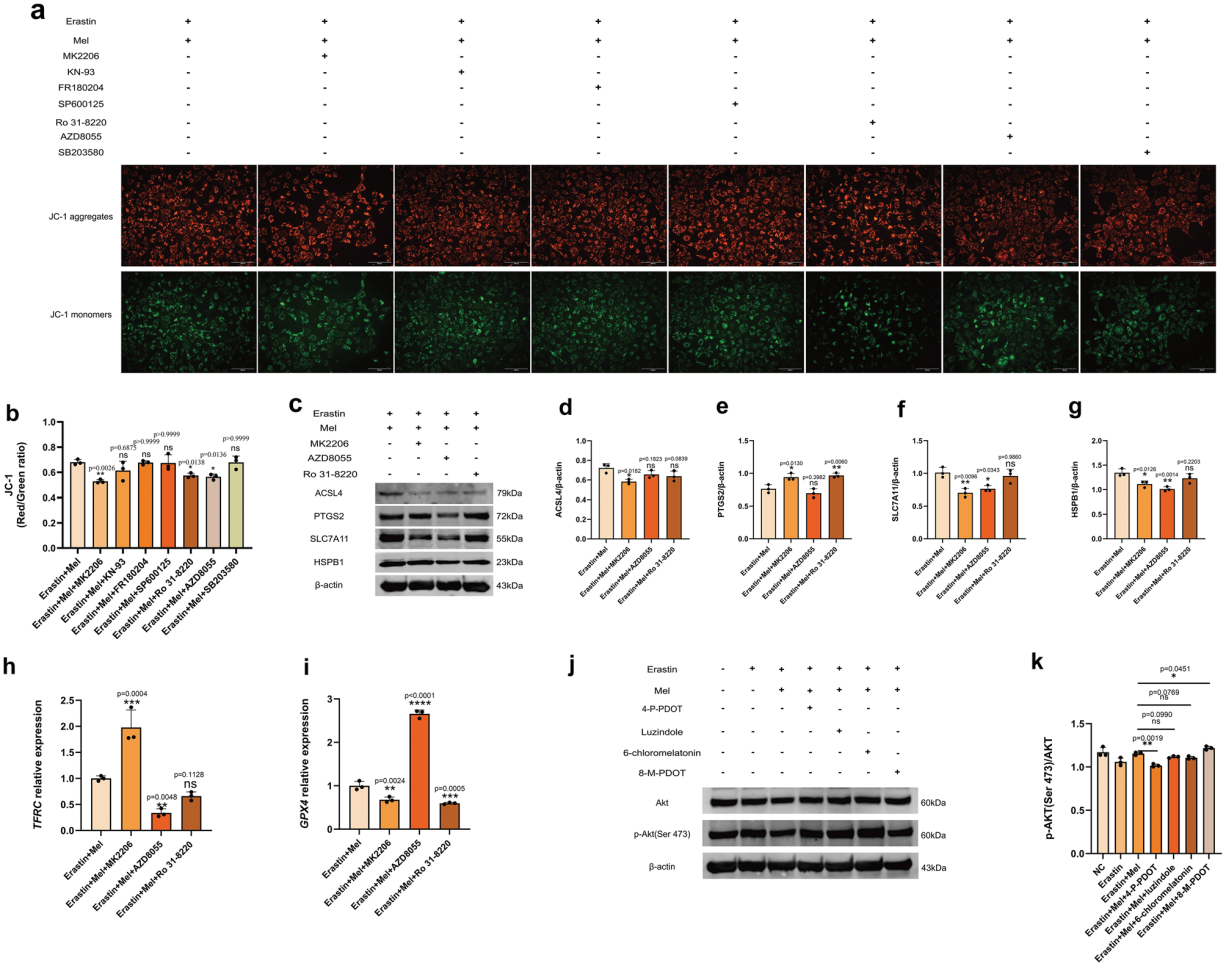
Melatonin exerts its protective effect against erastin-induced ferroptosis through Akt signaling pathway. (**a**) MMP were measured on ST cells. Cells were divided into eight groups: erastin + melatonin, erastin + melatonin + MK2206, erastin + melatonin + KN-93, erastin + melatonin + FR180204, erastin + melatonin + SP600125, erastin + melatonin + Ro31-8220, erastin + melatonin + AZD8055 and erastin + melatonin + SB203580. (**b**) Red/Green ratio of JC-1 staining. Mean fluorescence intensity was calculated by imagej. Statistical analysis between erastin + melatonin and other groups were using one-way ANOVA. (**c**) Western blot for ACSL4, PTGS2, SLC7A11, HSPB1 and β-actin were performed. ST cells were divided into four groups, erastin + melatonin, erastin + melatonin + MK2206, erastin + melatonin + AZD8055, erastin + melatonin + Ro 31–8220. Each group had three biological replicates. (**d**–**g**) Gray value analysis of ACSL4, PTGS2, SLC7A11 and HSPB1. Statistical analysis between erastin + Mel and others were analyzed using one-way ANOVA with three biological replicates per group. (**h**-**i**) Relative expression analysis results for *GPX4* and *TFRC*. One-way ANOVA was performed using the relative expression of three complex wells. (**j**) Western blot for Akt and p-Akt (Ser 473). Each group had three biological replicates. (**k**) Gray value analysis of p-Akt/Akt. The latter four groups were all analyzed for significance compared to erastin + melatonin group. All *P* values are annotated on the graph.*P < 0.05, **P < 0.01, and ***P < .001, *ns*, not significant.

The results showed that adding MK2206, an inhibitor of Akt, Ro31-8220, an inhibitor of PKC and AZD8055, an inhibitor of mTOR, all significantly decreased the MMP (Fig. 4a,b). To investigate which pathways are involved in the regulation of ferroptosis, we later examined the expression of ferroptosis biomarkers ACSL4, PTGS2, SLC7A11 and HSPB1 and found that inhibition of the Akt signaling pathway significantly resulted in the upregulation of PTGS2 expression and downregulation of ACSL4, SLC7A11 and HSPB1 expression (Fig. 4c–g), consistent with the trend changes when ferroptosis occurred. To provide further evidence, we performed qRT‒PCR for two ferroptosis biomarker genes, *GPX4*^30^ and *TFRC*, in which *TFRC* expression was observably upregulated and *GPX4* expression was evidently downregulated after inhibition of the Akt signaling pathway (Fig. 4h,i), providing further evidence that the Akt signaling pathway is involved in regulating Mel-eased erastin-induced ferroptosis. Finally, we examined the changes in p-Akt (Ser 473)/Akt after the addition of Mel receptor inhibitors and agonists. The results showed that the p-Akt/Akt ratio was significantly decreased after treatment with 4-P-PDOT and obviously increased after treatment with 8-M-PDOT (Fig. 4j,k). The abovementioned results confirmed that the Akt signaling pathway, as one of the downstream signaling pathways of MT2, is involved in mediating Mel to alleviate ferroptosis in ST cells.

### Mel affects mitochondrial-related protein expression through the MT2/Akt signaling pathway

**Figure 5:**
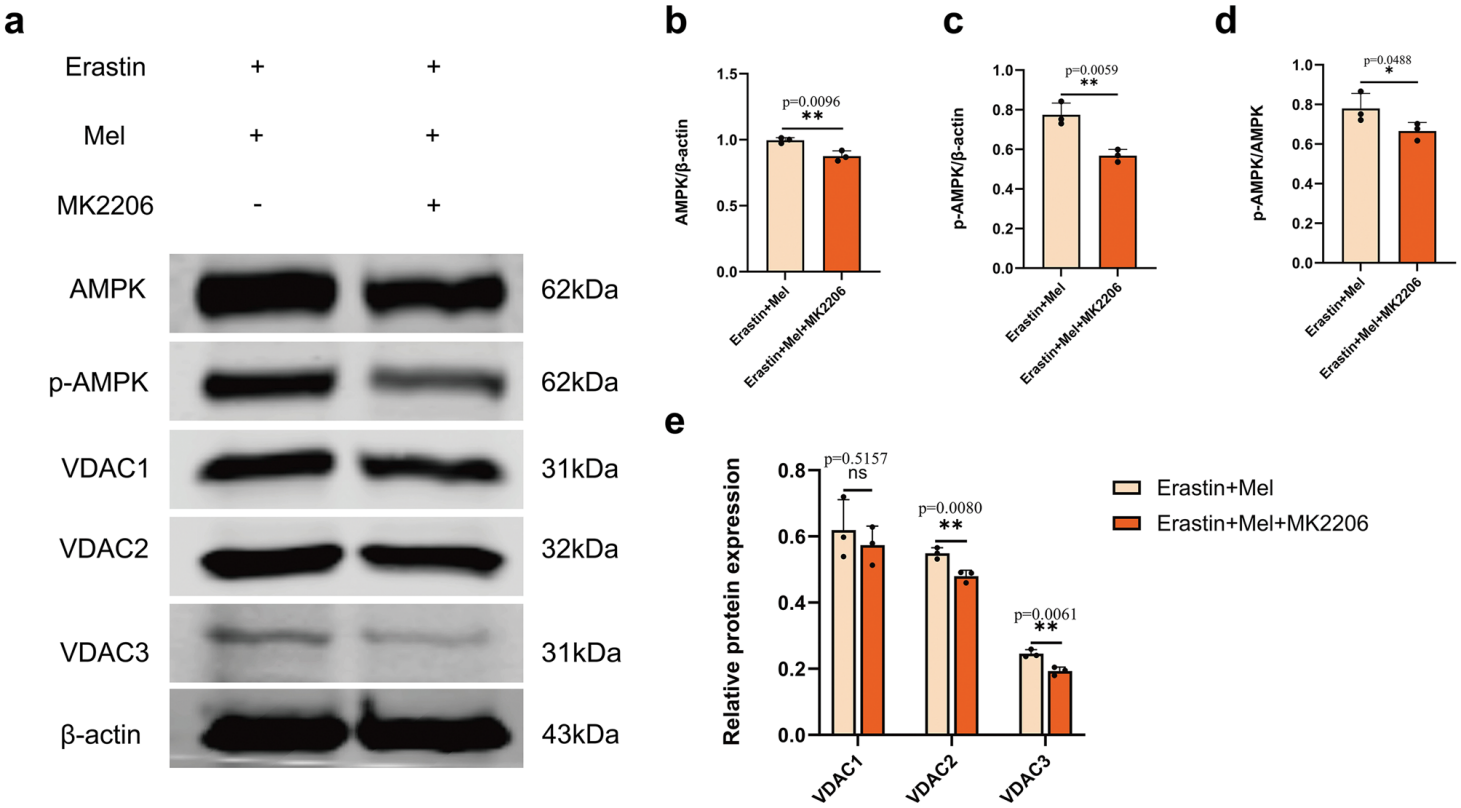
Akt signaling pathway affects the expression of key mitochondrial proteins. (**a**) Western blot for AMPK, p-AMPK, VDAC1, VDAC2, VDAC3 and β-actin were performed. ST cells were divided into erastin + melatonin and erastin + melatonin + MK2206. (**b**–**c**) Gray value analysis of AMPK and p-AMPK. (**d**) The ratio of p-AMPK/AMPK. (**e**) Gray value analysis of VDAC1, VDAC2 and VDAC3. Each group had three biological replicates, two-tailed t-test was used to detect differences between the groups and All *P* values are annotated on the graph .*P < 0.05, **P < 0.01, and ***P < 0.001, *ns*, not significant.

Adenosine 5'monophosphate-activated protein kinase (AMPK) is a cellular energy sensor that regulates the dynamic balance of mitochondrial energy metabolism, and it has also been shown that AMPK activation could inhibit ferroptosis^31^. To investigate whether inhibition of the Akt signaling pathway affects mitochondrial metabolic function, we examined the changes in AMPK and p-AMPK levels. The results indicated that inhibition of the Akt signaling pathway significantly decreased in AMPK, p-AMPK and p-AMPK/AMPK levels (Fig. 5a–d). To further investigate the effect of inhibition of the Akt signaling pathway on mitochondria-related proteins in ferroptotic cells, we examined the expression of the mitochondrial gated channel proteins voltage-dependent anion channel (VDAC). The results showed that inhibition of the Akt signaling pathway resulted in significant reductions in the expression of VDAC2 and VDAC3 (Fig. 5e), while the expression of VDAC1 was not affected. These results suggest that the beneficial effect of Mel on mitochondria subjected to ferroptosis is regulated by the MT2/Akt signaling pathway.

### Mel affects mtDNA expression and ATP content via the MT2/Akt signaling pathway

**Figure 6:**
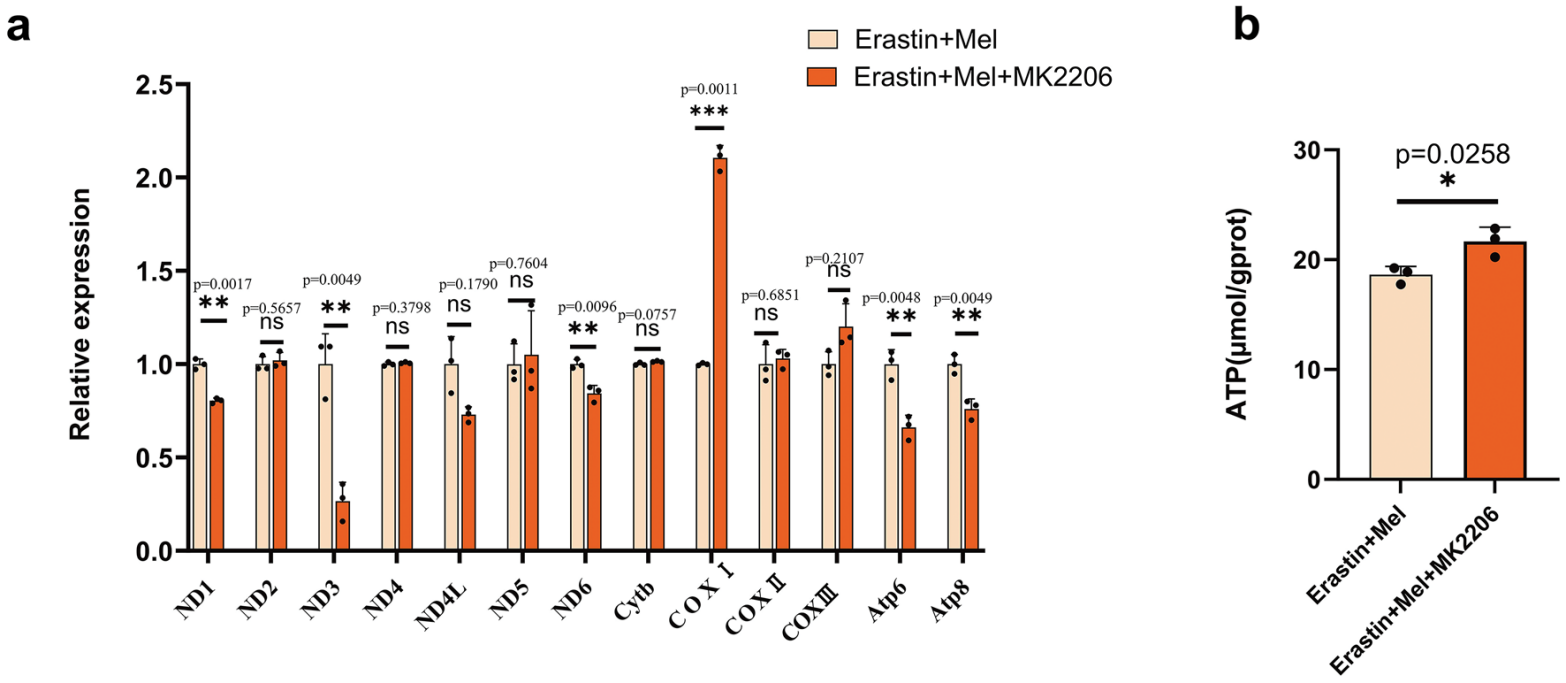
Akt signaling pathway affects the expression of mtDNA and ATP content. (**a**) Relative expression analysis results of mtDNA encoded subunits of the mitochondrial respiratory chain complex. Two-tailed t test was performed using the relative expression of three complex wells. (**b**) Detection of ATP content. ST cells were divided into erastin + melatonin and erastin + melatonin + MK2206. Two-tailed t test was performed to analysis the differences between two groups. Each group had three biological replicates and all *P* values are annotated on the graph *P < 0.05, **P < 0.01, and ***P < .001, *ns*, Not significant.

Mitochondria are the powerhouse of the cell, and their role in ferroptosis involves mtDNA biogenesis^32^. It was shown that zalcitabine-induced mtDNA depletion induces lipid peroxidation-mediated ferroptosis^33^. We therefore selected 13 genes encoding subunits of the mitochondrial respiratory chain complex for qRT‒PCR assays. The results revealed that inhibition of the Akt signaling pathway resulted in significant changes in the expression of 7 mitochondrial genes. Among the genes involved in encoding subunits of mitochondrial respiratory chain complex I, *ND1*, *ND3*, *ND4L* and *ND6* were significantly decreased (Fig. 6a). A significant increase in *COX I* occurred in the gene encoding subunits of mitochondrial respiratory chain complex IV, while *Atp6* and *Atp8*, both of which encode subunits of mitochondrial respiratory chain complex V, showed significant decreases (Fig. 6a). Finally, we further detected the ATP content in ST cells. The results showed that intracellular ATP content was markedly increased after inhibition of the Akt signaling pathway (Fig. 6b). The above results suggest that Mel affects mtDNA transcription and ATP content after the onset of ferroptosis via the MT2/Akt signaling pathway.

## Discussion

To the best of our knowledge, this is the first study that used erastin-induced ST cells to investigate whether Mel could alleviate testicular cell ferroptosis. Here, we provide evidence that Mel regulates mitochondrial functions to attenuate ferroptosis through MT2/Akt signaling pathway in ST cells.

The testis is an essential male reproduction organ and has been shown to be susceptible to ferroptosis^4,6,34,35^. Low doses of Cd cause decreased germ cell mass and abscission, decreased meiotic index and testicular weight and lead to testicular ferroptosis^36^. In the present study, we found that ferroptosis occurs after erastin induction in ST cells, which leads to a decrease in cell viability and cellular MMP, and an increase in LDH enzyme release. Mel as an important promote reproductive hormone, has been shown to be involved in regulating of male reproduction^37,38^. Studies have shown that Mel could regulate testosterone synthesis and testicular maturation^39^. The testis can also directly absorb Mel released from the pineal gland into the blood and regulate testicular activity^40^. Mel could alleviate the damage of pollutants on the testis, reduce the damage of testis caused by Cd and maintain good sperm quality^41^. Cd can cause ferroptosis, reduce testosterone production, and lead to testicular damage^36,41^. Meanwhile, clinical studies have shown that infertile men with reduced sperm motility and nonobstructive azoospermia exhibit abnormally low Mel levels^42^. We therefore hypothesized that Mel could alleviate ST cell ferroptosis. As we expected, the addition of Mel resulted in a significant increase in cell viability, a decrease in LDH enzyme release and an increase in MMP. TFRC, as an intracellular iron regulator, is upregulated by erastin stimulation, leading to intracellular iron accumulation and subsequent ROS generation^17^. Therefore, we measured the levels of ROS and intracellular ferrous iron content of ST cells. The results indicate that Mel alleviates the elevated ROS levels and ferrous iron accumulation caused by erastin. These results suggest that Mel may alleviate erastin-induced ferroptosis in ST cells.

To further determine whether Mel alleviates ferroptosis in ST cells, we inspected the expression of some ferroptosis biomarkers. It was shown that TFRC is elevated in ferroptosis-sensitive cells and that induction of TFRC expression leads to the occurrence of ferroptosis in cells^43,44^. PTGS2, also known as cyclooxygenase 2, a peroxidase involved in ferroptosis, is also significantly elevated after the occurrence of ferroptosis^21^. As a sodium-independent cystine-glutamate antiporter, system Xc- and its key component SLC7A11, previous studies suggest that overexpression of SLC7A11 inhibits ferroptosis to promote tumor growth^45^. NRF2 promotes SLC7A11 transcription and inhibition of NRF2 expression results in cells sensitive to ferroptosis^46^. HSPB1 is a negative regulator of ferroptosis, and knockdown of *HSPB1* enhances erastin-induced ferroptosis^19^. In our current study, we found that induced with erastin resulted in upregulation of TFRC, PTGS2, and downregulation of SLC7A11, NRF2 and HSPB1 expression in ST cells. In contrast, Mel reversed all of these changes, and the results further confirmed that Mel could alleviates ferroptosis in ST cells.

Mel generally acts in mammals through two G protein-coupled receptors, MT1 and MT2^47^, and MT2 have been shown to be involved in melatonin-regulated HT-22 cell ferroptosis^48^. In our study, inhibition of MT2 by 4-P-PDOT abated the effect of Mel on MMP and the impact on ACSL4, PTGS2 and HSPB1 expression. ACSL4 is an important isoenzyme in polyunsaturated fatty acid metabolism that determines sensitivity to ferroptosis^49^. The expression changes of these proteins indicated that Mel acts on the ST cells ferroptosis via MT2 receptor.

To investigate the downstream pathway of Mel improving erastin-mediated ferroptosis via MT2, the downstream signaling pathways of Mel/MT2 that appeared in the previous studies were selected for subsequent investigation. Studies have shown that Mel promotes the phosphorylation of PI3K/AKT through MT2 to improve intestinal dysfunction^50^; It mediates the increase of PKC activity and regulates circadian rhythm^51^. Mel could block the elevation of markers in dopaminergic neurons by activating MT2 and inhibiting CaMKII pathway^52^. Acute stimulation of MT2 resulted in the activation of ERK signaling pathway^25^. Mel protects endothelial cells from endoplasmic reticulum stress and mitochondrial dysfunction by inhibiting JNK/Mff signaling pathway^26^. Mel also reduced the phosphorylation of mTOR and inhibited milk fat synthesis^28^. It also inhibits cancer-associated osteoclast differentiation by downregulating the p38 MAPK pathway^29^. We found that inhibition of three pathways, Akt, mTOR and PKC, led to a markedly decrease in MMP. Meanwhile, inhibition of the Akt signaling pathway also resulted in upregulation of PTGS2 and TFRC and downregulation of ACSL4, SLC7A11, HSPB1 and GPX4. The results indicated that Akt signaling pathway was involved in the regulation of ferroptosis by Mel, and the Akt signaling pathway was downstream of Mel/MT2 by detecting p-Akt/Akt after adding agonists and inhibitors.

Akt serine-threonine kinase is involved in various physiological processes such as cell proliferation, metabolism, apoptosis and autophagy, and its overexpression is often seen in cancer^53^. It has been shown that upregulation of PI3K/Akt inhibits ferroptosis in non-small cell lung cancer cells^54^. Akt could directly phosphorylate SLC7A11, and Akt-mediated phosphorylation of SLC7A11 also inhibits its cystine transport activity, which in turn regulates ferroptosis^55^. Our results also revealed that SLC7A11 was sharply downregulated after Akt inhibition, suggesting that the expression of SLC7A11 was maybe regulated by Akt signaling pathway and associated with ferroptosis in ST cells.

AMPK is an enzyme that plays a role in intracellular environmental homeostasis, primarily activating glucose and fatty acid uptake and oxidation to maintain mitochondrial health. Studies have shown that inactivation of AMPK largely counteracts the protective effect of energy stress against ferroptosis in vitro and ferroptosis-related renal ischemia/reperfusion injury in vivo^56^. AMPK-activated cancer cells are resistant to ferroptosis, whereas blocked AMPK phosphorylation makes these cells sensitive to ferroptosis^57^. Our results show a decrease in AMPK expression after inhibition of the Akt signaling pathway, along with a decrease in phosphorylated AMPK, representing that ST cells may be more sensitive to ferroptosis after inhibition of the Akt signaling pathway. VDAC are key mitochondrial protein that controls cell life and death and has three isoforms VDAC1, VDAC2 and VDAC3^58^. VDACs mainly participate in cell energy metabolism by affecting the transport of Adenosine Triphosphate (ATP)or Adenosine diphosphate (ADP) inside and/or outside the mitochondria^59^. VDAC2/3 is localized in the outer membrane of mitochondria and regulates the movement of numerous ions and metabolites^60^. Deletion of VDAC2/3 affects mitochondrial activity by disrupting the balance of different ions and metabolites^61^. Our results showed no obvious change in VDAC1 expression after inhibition of the Akt signaling pathway, but both VDAC2 and VDAC3 were significantly reduced. Mel may affect the expression of VDAC2/3 through the Akt signaling pathway, which in turn regulates the permeability of the outer mitochondrial membrane and further attenuates the onset of cellular ferroptosis.

Mitochondria are bilayer membrane organelles in eukaryotic cells that undergo oxidative phosphorylation and produce energy in the form of ATP. Through electron-transport chain complexes located in the inner membrane of the mitochondria, including complexes I, II, III, and IV, electrons are transferred from the electron donor to the electron acceptor and eventually to oxygen. The electron transport chain combines with a proton pump from the mitochondrial matrix into the intermembrane compartment to establish proton dynamics that ultimately drive ATP synthesis via complex V^62^. Mitochondrial DNA has 13 coding genes that encode proteins constituting the subunits of the respiratory chain protein complexes. We then selected these genes for qRT‒PCR assays. Our results showed that inhibition of the Akt signaling pathway significantly decreased in *ND1*, *ND3*, *ND6*, *Atp6* and *Atp8*. *ND1*, *ND3* and *ND6* are involved in encoding mitochondrial complex I (ubiquinone oxidoreductase), which is the largest protein complex in the inner mitochondrial membrane, possibly indicating that the Akt signaling pathway affects complex I activity and mitochondrial electron transport by affecting mtDNA transcription. Atp6 and Atp8 are involved in encoding mitochondrial complex V, which catalyzes the synthesis of ATP using an electrochemical gradient of protons generated by the respiratory chain^63,64^. However, our results indicate that the ATP content was elevated after inhibition of the Akt signaling pathway. The reason may be due to the decreased expression of Atp6 and Atp8, which do not affect the activity of mitochondrial complex V, but the exact molecular mechanism needs to be further investigated.

**Figure 7:**
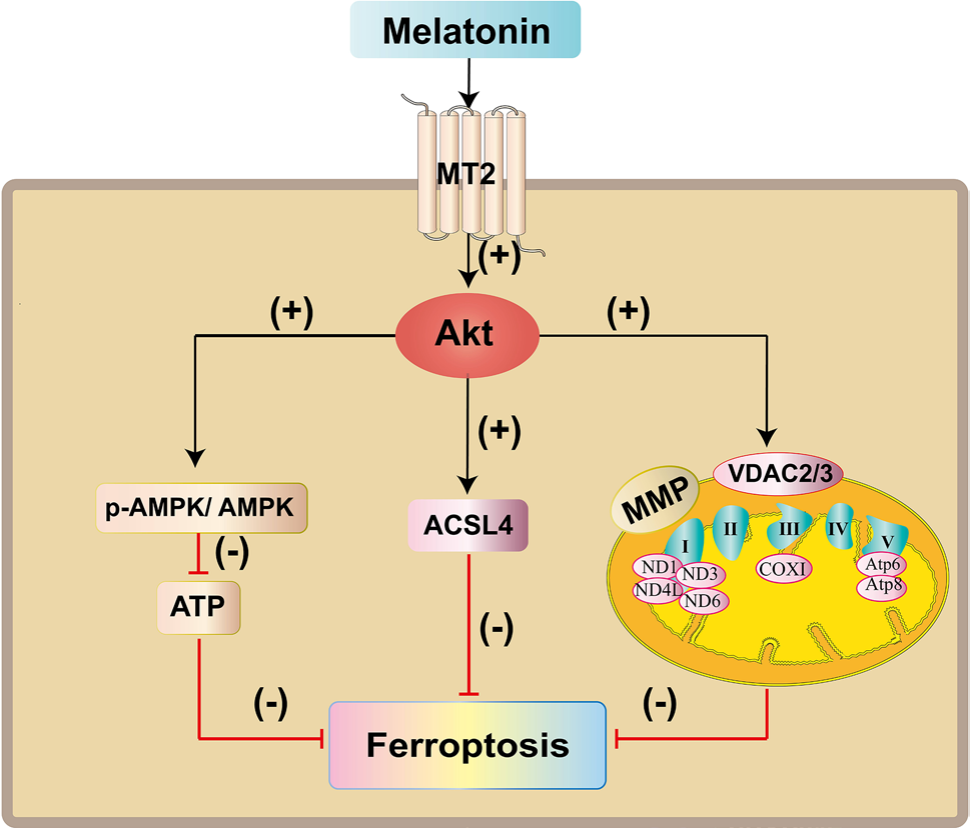
Schematic representation of the protective mechanism of melatonin against erastin-induced ferroptosis in ST cells. Exogenously added melatonin inhibits ferroptosis of ST cells through Akt signaling pathway after binding to MT2. Akt signaling pathway is activated, then regulates AMPK phosphorylation, ACSL4 expression, affects mitochondrial function (MMP, VDAC2/3 expression, mtDNA transcription, intracellular ATP content), further inhibits erastin-induced ferroptosis in ST cells.

Taken together, our results suggest that Mel alleviates ST cells from damage caused by erastin-induced ferroptosis and acts through MT2. In the downstream pathway of MT2, Mel affects MMP, the expression of mitochondria-related proteins, the transcription of genes encoding mitochondrial subunits and ATP content through the Akt signaling pathway and further inhibits erastin-induced ferroptosis in ST cells (Fig. 7).

## Conclusion

The present study provides insights into the biological functions of Mel and suggests that exogenous addition of Mel can binding to MT2 then regulate mitochondrial function and reduce ferroptosis in ST cells through Akt signaling pathway.

## Materials and methods

### Drug administration

Mel was dissolved in dimethyl sulfoxide (DMSO) to a final concentration of 1 mM. The dosage of Mel was determined by the MTT method. Luzindole, an antagonist of MT1 and MT2, and 4-P-PDOT, an antagonist of MT2, were both dissolved in DMSO and then administered at 10 µM 30 min before the addition of Mel. 6-Chloromelatonin, an agonist of MT1, and 8-M-PDOT, an agonist of MT2, were also dissolved in DMSO and then administered at 100 µM while we added Mel. All chemicals used in this study were purchased from Sigma‒Aldrich Chemical Company (St. Louis, MO, USA) unless otherwise stated.

### Cell culture

ST cells were purchased from American Type Culture Collection (Virginia, USA). ST cells were cultured in T25 flask containing 5mL DMEM (HyClone, Logan, USA) with 10% fetal bovine serum (HyClone) and 1% penicillin/streptomycin (Beyotime, Shanghai, China). ST cells were incubated at 37 ℃ with 5% CO_2_. After 2 days of culture, the cells were digested with 0.25% trypsin and then centrifuged at 1000 rpm for 5 min. The supernatant was discarded, and the cells were maintained in complete cell culture medium and cutured at 37 ℃ with 5% CO_2_.

### Cell viability assay

To verify whether Mel has a protective effect against erastin-induced ferroptosis, ST cells were seeded into 96-well plates (8 × 10^3^ per well), cultured for 12 h, treated with 10 µM erastin, and incubated for 24 h. Then, the cells were incubated with 150 µM Mel to evaluate cell viability. Cell growth and viability were measured using MTT. Briefly, MTT (20 µL) was added to 80 µL of DMEM in each well and incubated for 4 h at 37 ℃. The contents were centrifuged, and the supernatant was carefully aspirated out and replaced with 150 µL of DMSO. Absorbance was then measured at 490 nm using a microplate reader (Thermo, Waltham, USA), and cell viability presented as OD values of experimental wells/control wells.

### LDH release assay

Intracellular LDH release was determined with an LDH cytotoxicity assay kit (Beyotime). Briefly, after ST cells were treated with different treatments, the supernatants were harvested. LDH reagent was used to detect LDH release at a wavelength of 490 nm according to the instructions.

### Mitochondrial membrane potential detection

Mitochondrial membrane potential (MMP) was detected with a JC‐1 Staining Kit (Beyotime). At high MMP, JC-1 was aggregated in the mitochondrial matrix and formed J-aggregates, which could produce red fluorescence. At low MMP, JC-1 could not accumulate in the matrix of mitochondria, and JC-1 was a monomer and could produce green fluorescence. The relative ratio of red and green fluorescence is commonly used to measure the proportion of mitochondrial depolarization. Cells from different groups were first washed with PBS. Then, the cells were incubated with a 1 mL mixture of JC‐1 working solution in the dark for 30 min. Then, 8 mL of ultrapure water per 50 µL of JC-1 (200 ×) was added and mixed well, and 2 mL of JC-1 staining buffer (5 ×) was added and mixed to make the JC-1 working solution. Then, the cells were washed with cold JC-1 staining buffer twice. For every 1 mL of JC-1 staining buffer (5 ×), add 4 mL of distilled water to prepare the appropriate amount of JC-1 staining buffer (1 ×). Afterward, the MMP of different samples was detected by fluorescence microscopy (Nikon, Tokyo, Japan). The ratio of red fluorescence intensity to green fluorescence intensity was analyzed with ImageJ software. The experiment was repeated three times, each time three visual fields were selected for analysis and the average value was calculated.

### Detection of intracellular ROS concentration

ST cells were added to 6-well plates. After collection, ST cells were resuspended in DMEM containing 10 μM/L DCFH-DA (Beyotime), incubated at 37℃ for 30 min, inverted every 3-5 min, and washed with DMEM for three times. The mean fluorescence value of FITC was measured using BD Accuri C6 Plus (New Jersey, USA). Flowjo 10.6.2 was used to process fcs data.

### Detection of intracellular ferrous iron content

Intracellular ferrous iron content was determined with an iron assay kit (BioVision). Briefly, after ST cells were treated with different treatments, Resuspend the cells in iron assay buffer, then incubate the test samples and iron standards at 25 °C for 30 min. After the incubation, add iron ion probe to each well, gently mix. Allow the reaction to proceed in the dark at 25 °C for 1 h. Subsequently, read the absorbance at 593 nm.

### Quantitative determination of total protein

After the cell homogenate was extracted, 10 µL of the dilution was used to determine the total protein content using the total protein assay kit (with standard: BCA method) (Nanjing Jiancheng, Nanjing, China). Specific procedures were performed according to the manufacturer's instructions. The total protein content was calculated according to the manufacturer's instructions based on the absorbance at 562 nm.

### Western blotting

Cells were lysed in RIPA lysis buffer (Beyotime) containing 1 mM PMSF (Beyotime). For 6-well plates, we used 200 µL RIPA lysis and centrifugation at 13,000 × g for 15 min, and the supernatant was collected. Then, 6 × protein loading buffer (TransGen Biotech, Beijing, China) was added at a ratio of 1:5, incubated in a water bath at 100 °C for 10 min and stored at -20 °C. Proteins (20 µL-40 µL) were separated by SDS‒PAGE using 10% and 12% gels. Proteins were transferred onto nitrocellulose membranes (Pall Corporation, New York, USA) and blocked in 5% skim milk (Sangon Biotech, Shanghai, China) in Tris-buffered saline (TBS) for 2 h at room temperature. The membranes were incubated with primary antibodies (Table S1) against TFRC, PTGS2, NRF2, SLC7A11, HSPB1, Akt, p-Akt (Ser 473), AMPK, p-AMPK, VDAC1, VDAC2, and VDAC3 overnight at 4 ℃. After three 5-min washes in TBST, the membranes were incubated with secondary antibodies (Table S1) for 1 h at room temperature, and proteins were visualized and analyzed using a LI-COR Odyssey Infrared Fluorescent System (Nebraska, USA).

### RNA extraction and reverse transcription

Cells were gently washed with sterile PBS three times, and total RNA extraction was performed by using NucleoZOL reagent (Genecompany, Wuhan, China) according to the manufacturer's instructions. The RNA integrity and concentration were determined by 1% agarose gel and Epoch Microplate Spectrophotometer (Biotek, Vermont, USA). One microgram of qualified RNA per sample was used for cDNA synthesis by a FastKing cDNA First-Strand Synthesis Kit (Tiangen, Beijing, China).

### Quantitative real-time PCR analysis

Quantitative real-time PCR (qRT‒PCR) analysis was performed to validate the expression profile of different genes, and GAPDH was used as an internal control. The primers used in qRT‒PCR are summarized in Table 1. Among them, 13 mitochondrial coding gene primers were referenced from the study of Zhu et al.^65^. qRT-PCRs were performed using the FastKing One-Step Reverse Transcription Fluorescence Quantitation Kit (Tiangen). All qRT‒PCR assays were performed in duplicate on a Roche LightCycler 480 (Roche, Basel, Swiss). All qRT‒PCR experiments were performed in triplicate using independent samples. The expression levels were determined by the 2^-∆∆Ct^ method.

**Table 1:** Primers used for qRT-PCR validation of differentially expressed genes.

Gene name	Forward primer (5′-3′)	Reverse primer (5′-3′)	Size (bp)
GPX4	TGTGGTTTACGGATTCTGG	CCTTGGGCTGGACTTTCA	181
TFRC	GTTGAACAGAATGGCACG	CTCGGAGATACATAGGGT	333
ND1	AATATGGCGAAAGGTCCGGC	ACCCTAGCAGAAACCAACCG	104
ND2	TGGCTAGGGCCATGGTTATT	CCTAACACAAGCCACAGCCT	152
ND3	GAGGCCTGCTGATCCTATCG	AACCCTAGCCTCCCTACTCG	130
ND4	AGGAGTGTTTGCAGTCCTCG	TGCCCACGGACTAACATCC	149
ND4L	AGCTAGGGTGAAGTGTGTGT	GATCGCCCTTGCAGGGTTAC	127
ND5	GAAGGCGTAGGATACGGTGG	CCCATTCGCCTCACTCACAT	154
ND6	AAGCAGCAATCCCCATAGCTT	GCGTTGAAGGAAGAGGAAGTAGA	118
Cytb	TAGGGCCAACACTCCACCTA	CACCCCAGCAAACCCACTAA	115
CoxI	ACAGTTCATCCAGTACCCGC	TCCCGATATGGCCTTTCCAC	169
Cox II	GGCATGAAGCTGTGGTTTGA	GATGCTATCCCAGGACGACT	108
Cox III	ATACTCCTGAGGCGAGGAGG	CCTAGCACCAACACCCGAAT	132
Atp6	TTGGATCGAGATTGTGCGGT	TGCCCCCACGATAATAGGAC	188
Atp8	ATACCCAGCAAGCCCAGAAT	GTGGGGGCAATAAAAGAGGCA	115
GAPDH	GAGATCCCGCCAACATCAA	TTCACGCCCATCACAAACA	169

### Detection of ATP level

Intracellular ATP content was measured using an ATP assay kit (Nanjing Jiancheng). ST cells were added to 6-well plates, treated with erastin, Mel and MK2206, digested and centrifuged. After removal of the supernatant, the cell suspension was homogenized with cold distilled water, and then the cell suspension was boiled for 10 min and vortexed for 1 min. The total ATP content was then determined according to the manufacturer's instructions. The total amount of ATP was calculated from the absorbance at 636 nm and the protein content measured by BCA.

### Statistical analysis

Data are presented as the mean ± SEM. Statistical analyses were performed using GraphPad Prism 10.1.2 Software (San Diego, USA). Data were analyzed by t test between two groups. ANOVA (one-way) followed by Dunnett's post hoc test was used to analyzed multiple groups. Data are presented as the mean ± SD of three independent repeats. P < 0.05 was considered to indicate a statistically significant difference.

### Supplementary Information

Supplementary Information 1.

Supplementary Information 2.

## Data Availability

Data is provided within the manuscript or supplementary information files.
