# Supplementary material for: Melatonin regulates mitochondrial function to alleviate ferroptosis through the MT2/Akt signaling pathway in swine testicular cells
Source: Sci Rep. 2024 Jul 2;14:15215. doi: 10.1038/s41598-024-65666-1 (PMC11219911; doi:10.1038/s41598-024-65666-1)

Figure 2A The original gel of TFRC from ST cells.

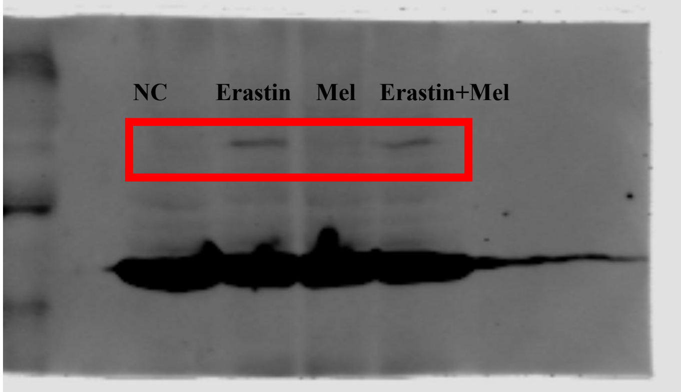

Figure 2A The original gel of PTGS2 from ST cells.

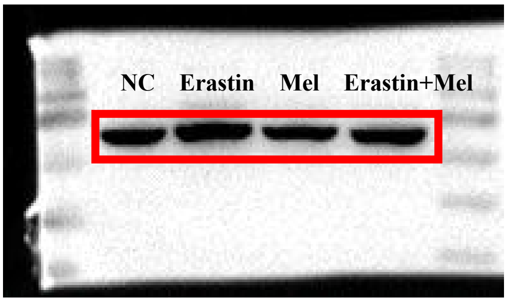

Figure 2A The original gel of NRF2 from ST cells.

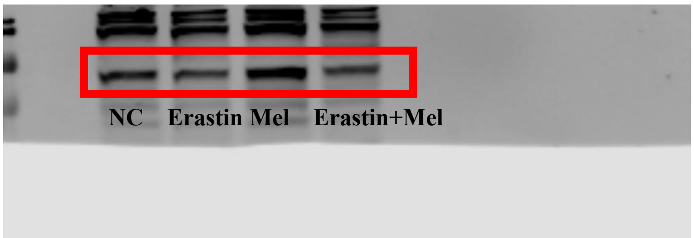

Figure 2A The original gel of SLC7A11 from ST cells.

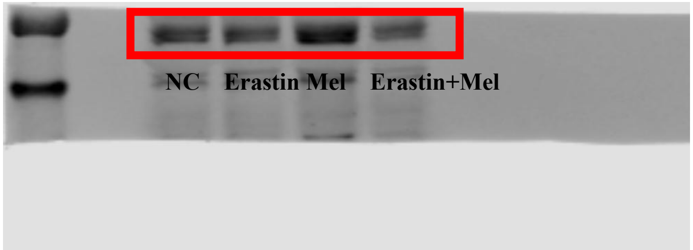

Figure 2A The original gel of HSPB1 from ST cells.

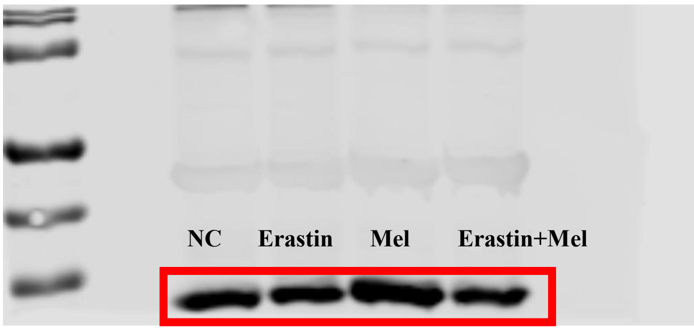

Figure 2A The original gel of β-actin from ST cells.

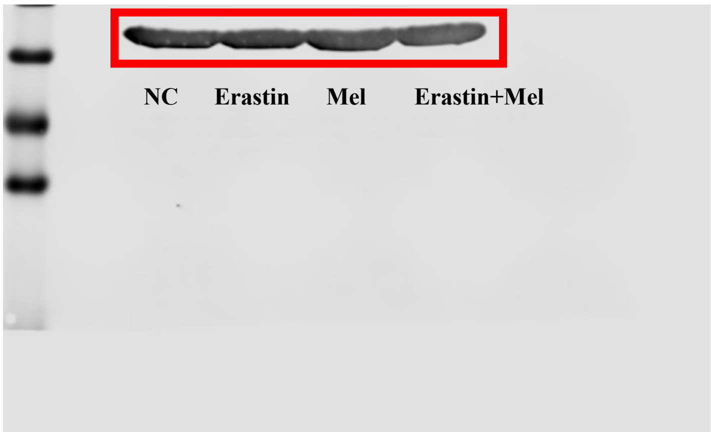

Figure 3C The original gel of ACSL4 from ST cells.

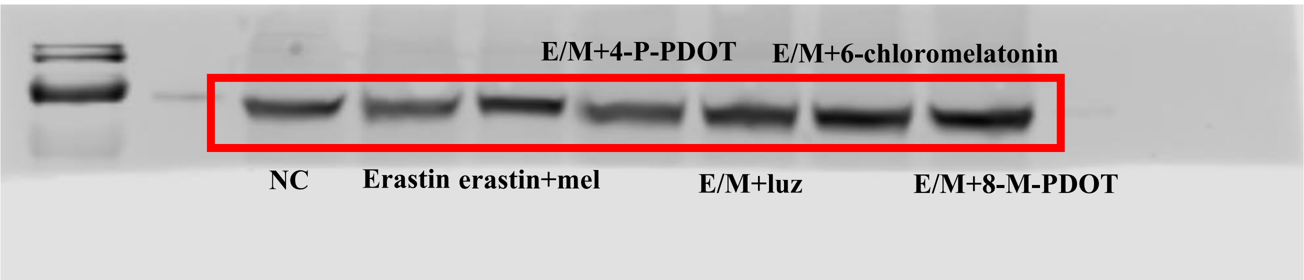

Figure 3C The original gel of PTGS2 from ST cells.

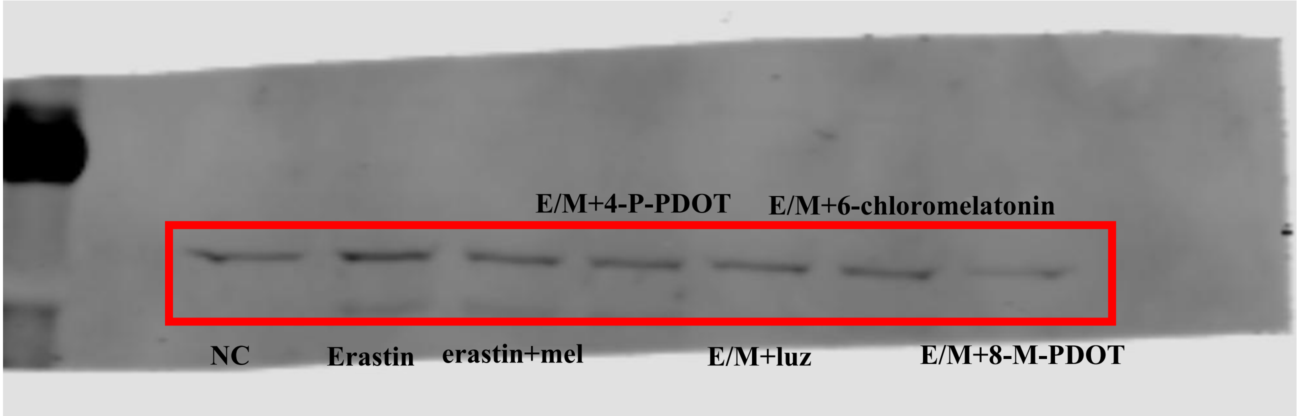

Figure 3C The original gel of HSPB1 from ST cells.

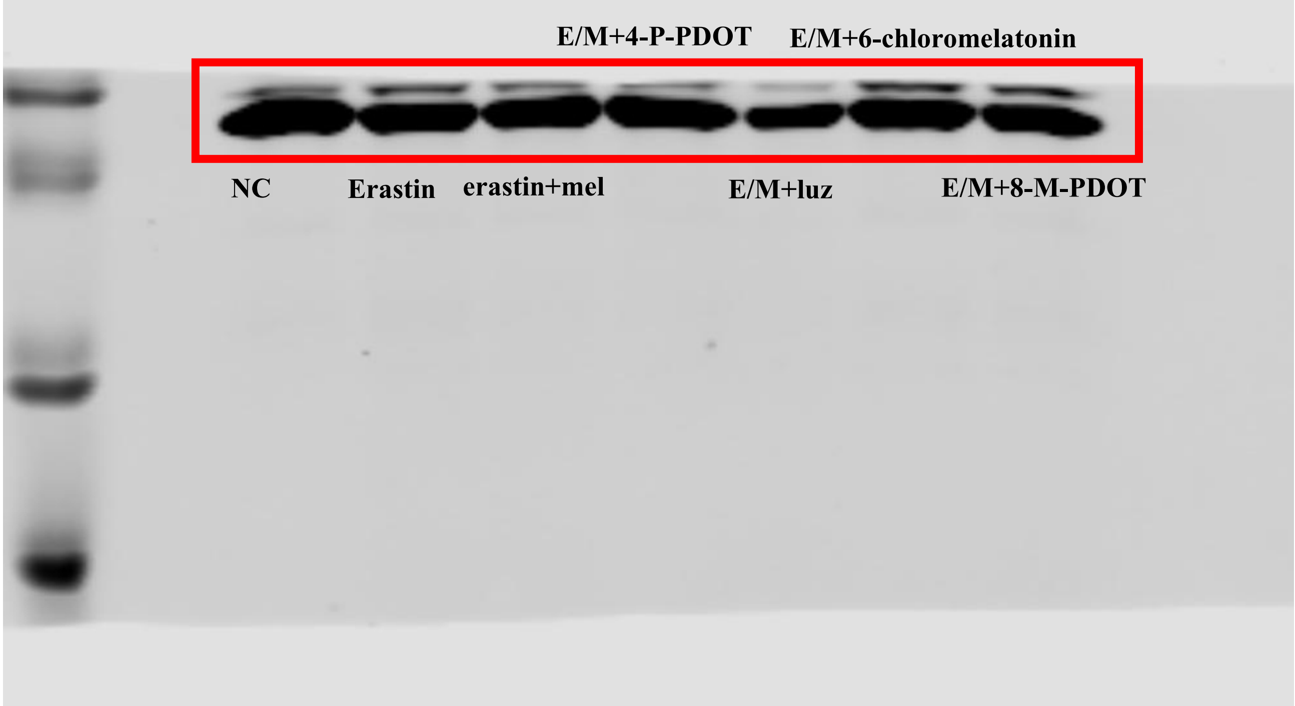

Figure 3C The original gel of β-actin from ST cells.

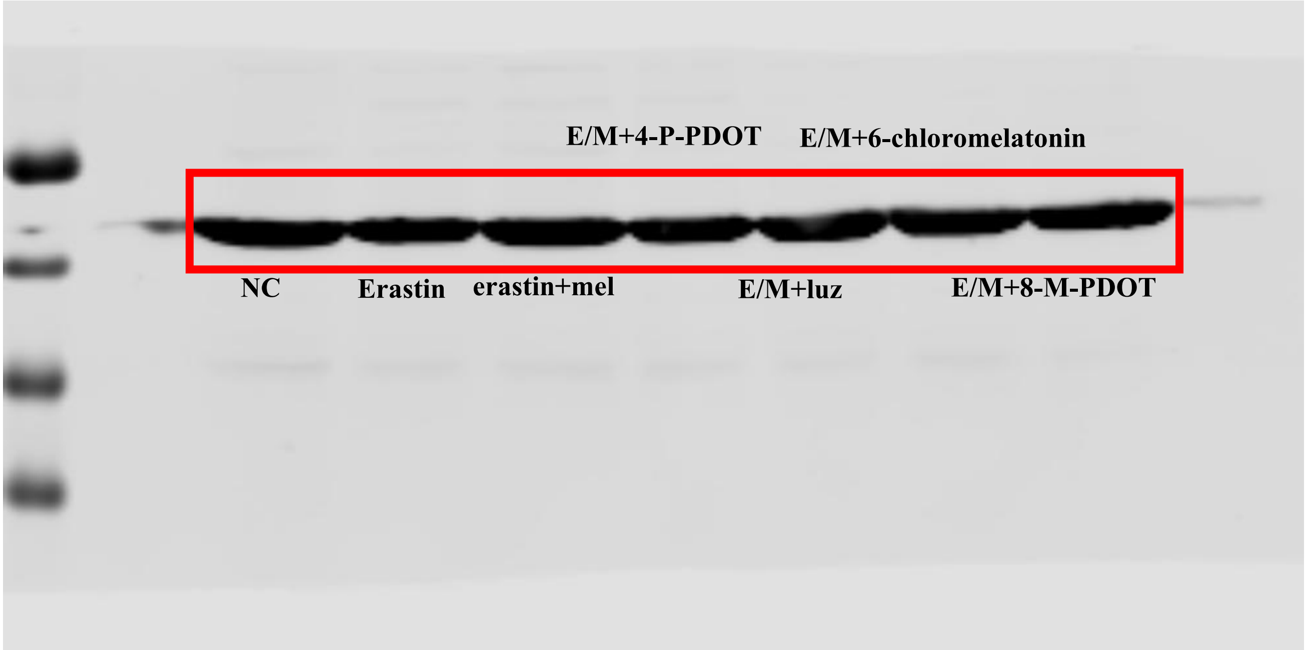

Figure 4C The original gel of ACSL4 from ST cells.

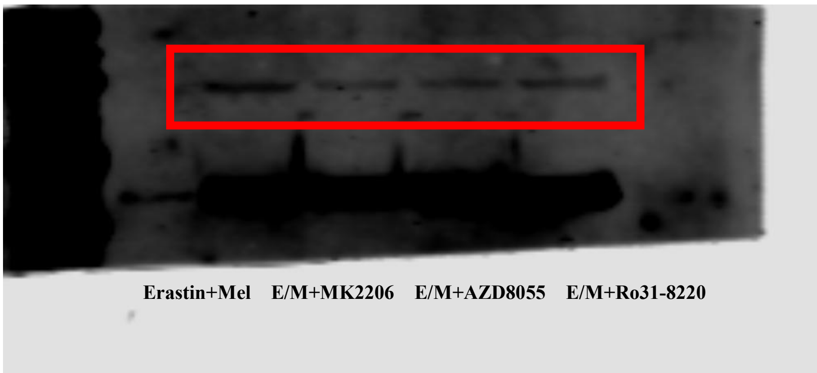

Figure 4C The original gel of PTGS2 from ST cells.

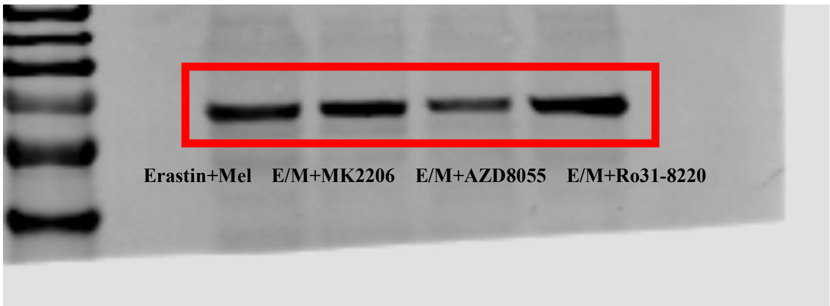

Figure 4C The original gel of SLC7A11 from ST cells.

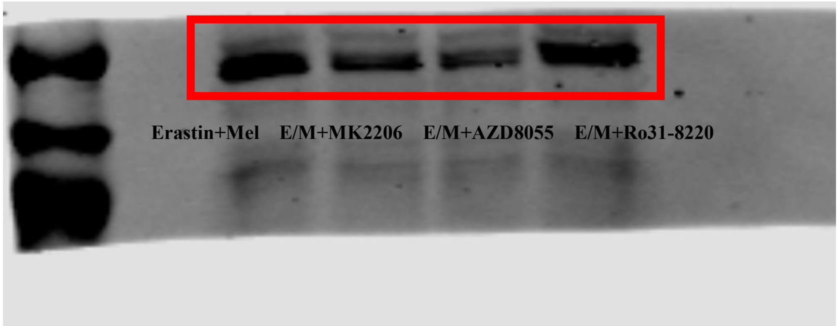

Figure 4C The original gel of HSPB1 from ST cells.

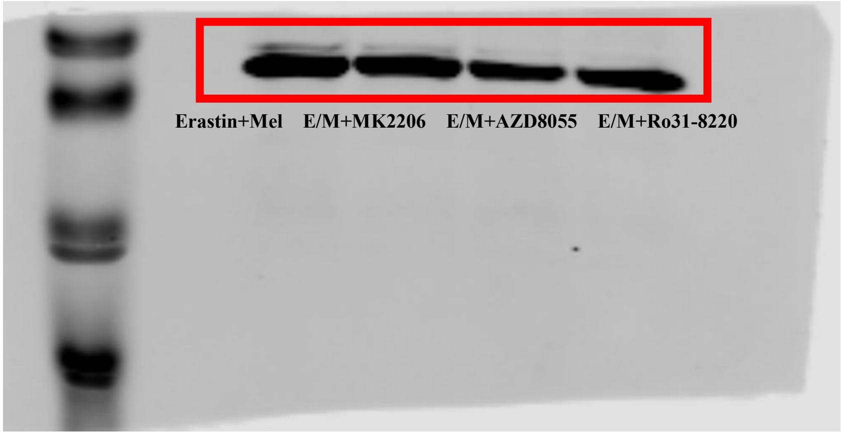

Figure 4C The original gel of β-actin from ST cells.

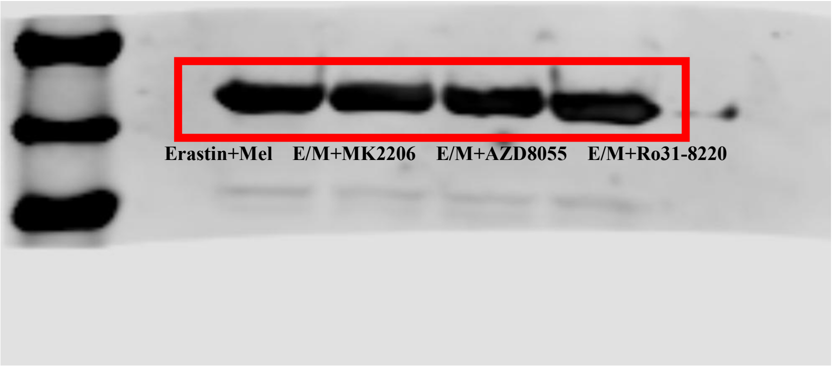

Figure 4J The original gel of Akt from ST cells.

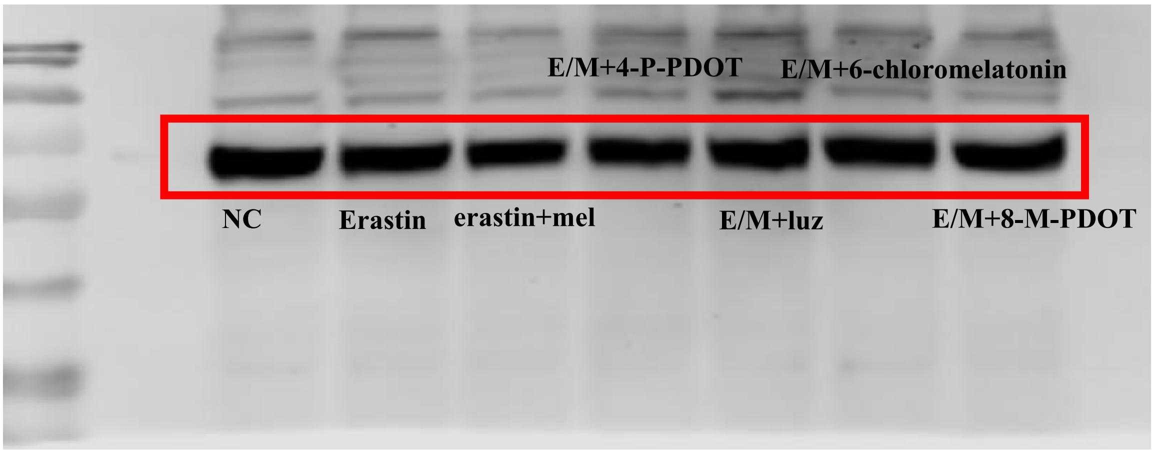

Figure 4J The original gel of p-Akt from ST cells.

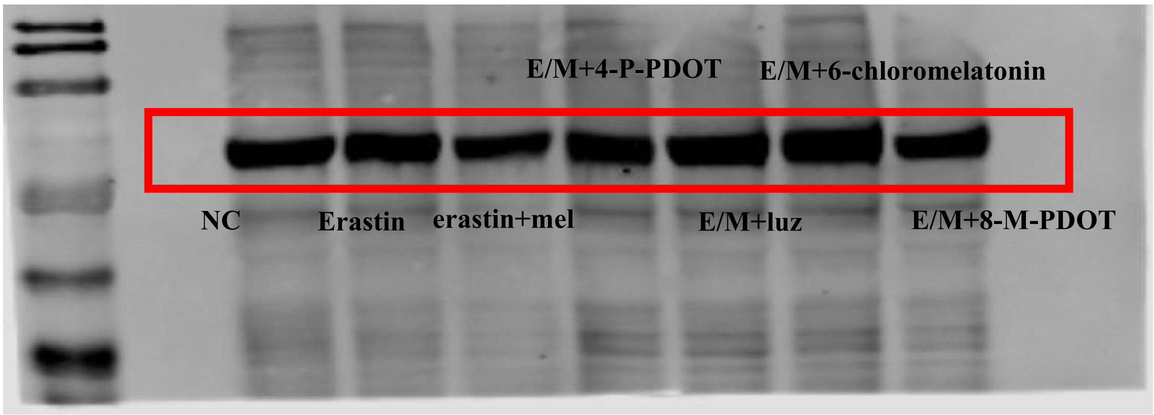

Figure 4J The original gel of β-actin from ST cells.

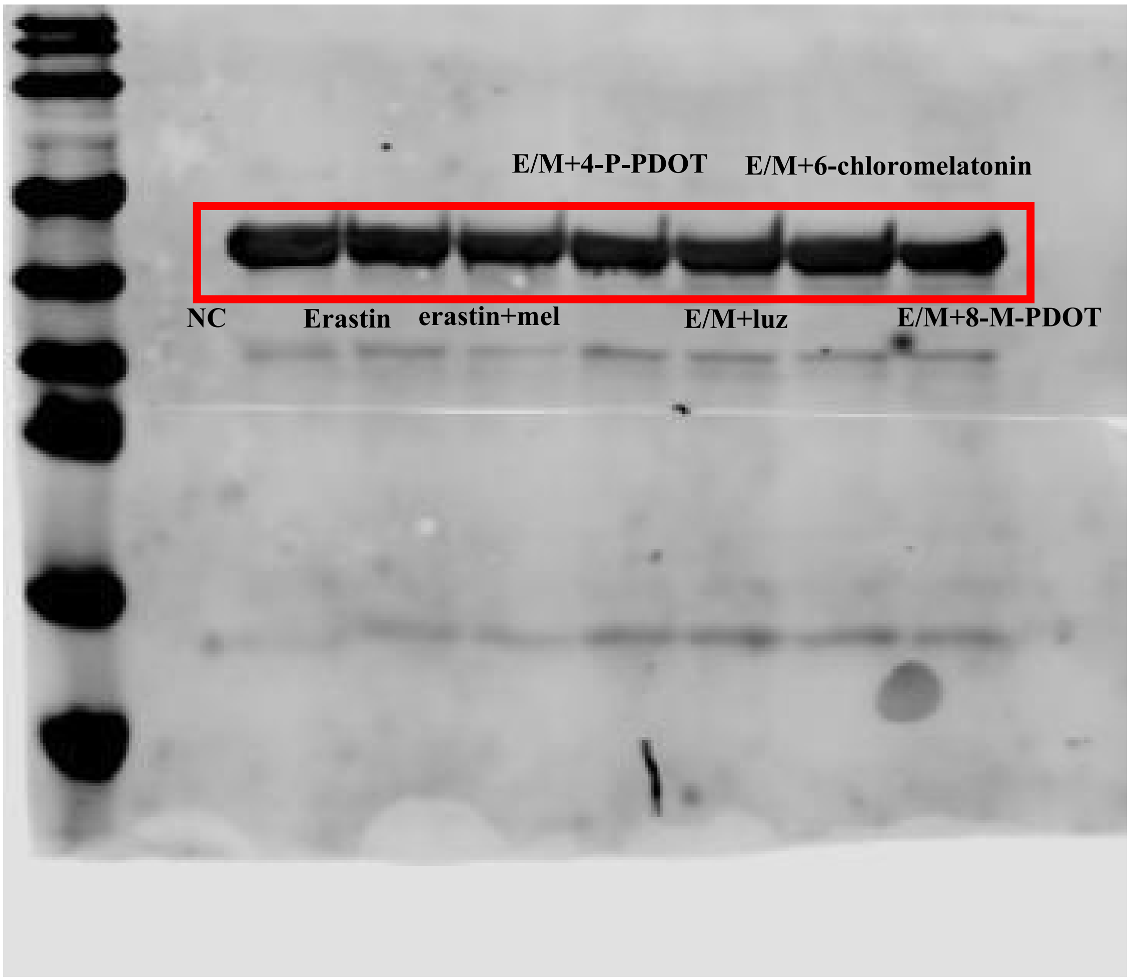

Figure 5A The original gel of AMPK from ST cells.

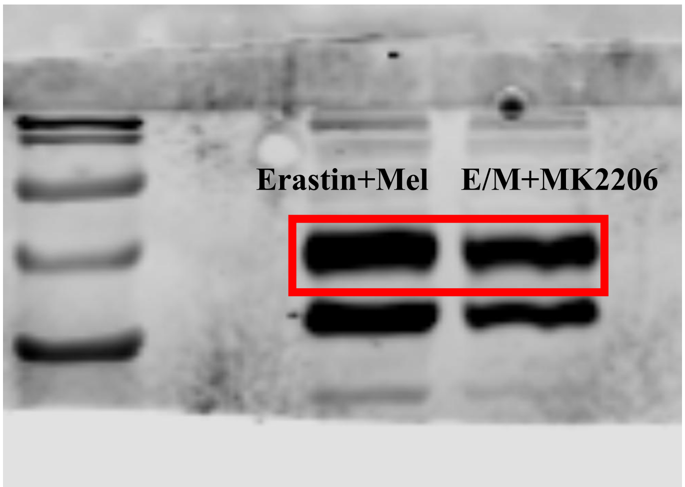

Figure 5A The original gel of p-AMPK from ST cells.

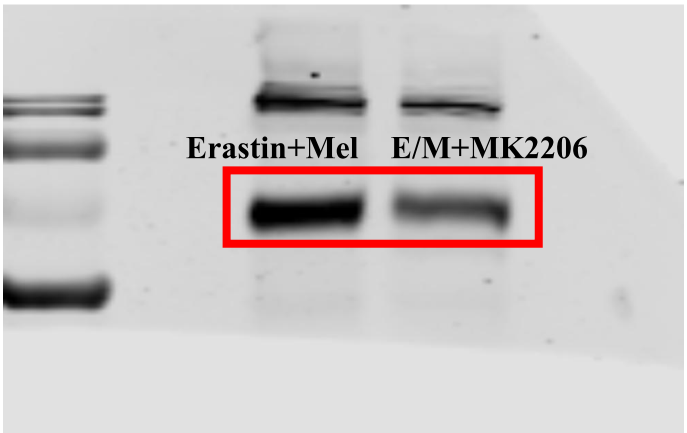

Figure 5A The original gel of VDAC1 from ST cells.

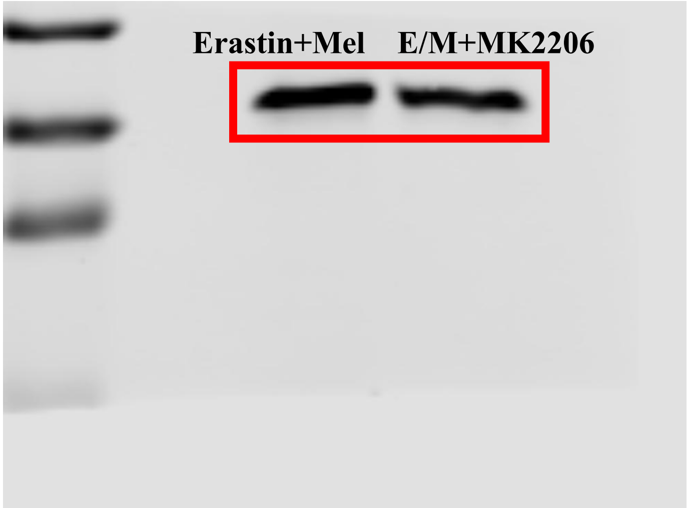

Figure 5A The original gel of VDAC2 from ST cells.

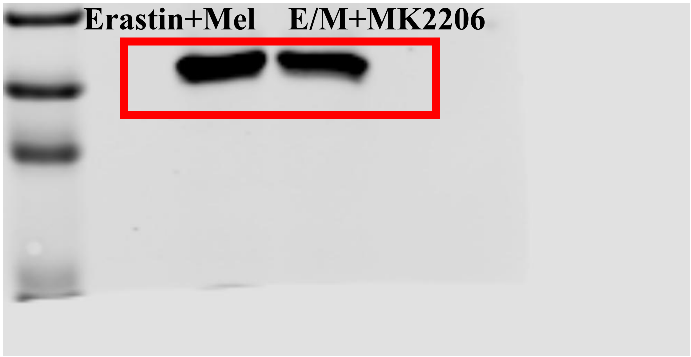

Figure 5A The original gel of VDAC3 from ST cells.

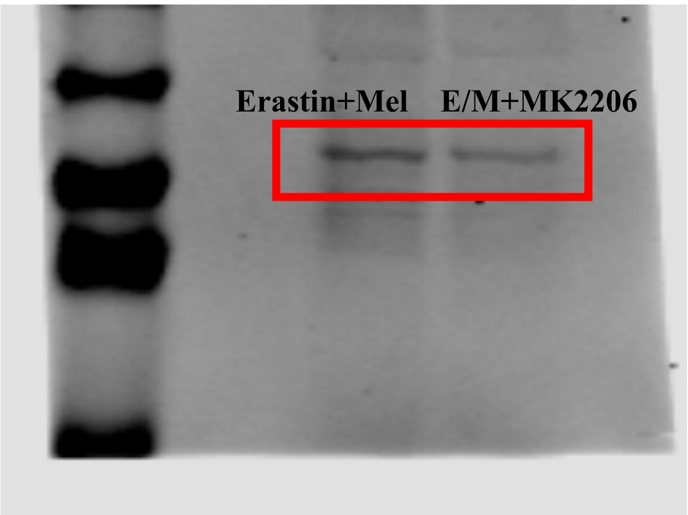

Figure 5A The original gel of β-actin from ST cells.

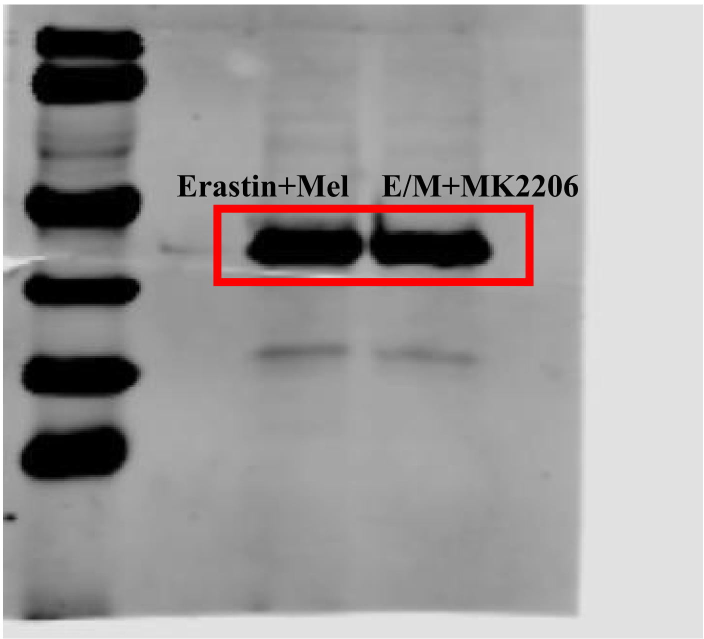

Supplement: Supplementary file 1 — Supplementary Information 1. [file 41598_2024_65666_MOESM1_ESM.pdf]
