# Supplementary material for: Melatonin regulates mitochondrial function to alleviate ferroptosis through the MT2/Akt signaling pathway in swine testicular cells
Source: Sci Rep. 2024 Jul 2;14:15215. doi: 10.1038/s41598-024-65666-1 (PMC11219911; doi:10.1038/s41598-024-65666-1)
Supplement: Supplementary file 2 — Supplementary Information 2. [file 41598_2024_65666_MOESM2_ESM.docx]

**Supplementary table**

Table S1 Primary and secondary antibodies used for Western blotting

| **Antibody** | **Host** | **Distributor** | **Working dilution** |
| --- | --- | --- | --- |
| **Primary antibody** |  |  |  |
| β-actin | M | TransGen Biotech HC201-01 | 1:10000 |
| TFRC | M | Santa Cruz (sc-32272) | 1:500 |
| PTGS2 | M | Santa Cruz (sc-19999) | 1:500 |
| NRF2 | R | Sangon Biotech (D221053) | 1:1000 |
| SLC7A11 | R | Sangon Biotech(D262619) | 1:1000 |
| HSPB1 | R | Sangon Biotech (D155223) | 1:1000 |
| Akt | R | Beyotime (AA326) | 1:1000 |
| P-Akt (Ser 473) | R | Beyotime (AA329) | 1:1000 |
| AMPK | R | Proteintech (10929-2-AP) | 1:1000 |
| p-AMPK | R | AFFIITY (AF3423) | 1:1000 |
| VDAC1 | R | Sangon Biotech (D151112) | 1:1000 |
| VDAC2 | R | Sangon Biotech (D151113) | 1:1000 |
| VDAC3 | R | Sangon Biotech (D223531) | 1:1000 |
| **Secondary antibody** |  |  |  |
| IRDye 680CW Donkey anti-Rabbit IgG | D | LI-COR (926-68073) | 1:10000 |
| IRDye 800CW Goat anti-Mouse IgG | G | LI-COR (926-32210) | 1:10000 |
| IRDye 800CW Goat anti-Rabbit IgG | G | LI-COR (926-32211) | 1:10000 |
|  |  |  | |

Notes: M: Mouse; R: Rabbit; D: Donkey; G: Goat
